# High-Impact Pain Predicts Incidence of Subjective and Objective Cognitive Decline

**DOI:** 10.21203/rs.3.rs-6149682/v1

**Published:** 2025-03-26

**Authors:** Javier Tamargo, Glenn Smith, Li Chen, Yenisel Cruz-Almeida

**Affiliations:** University of Florida; 1Florida Alzheimer’s Disease Research Center; University of Florida

## Abstract

Chronic pain is the most common health challenge for older adults and a significant risk factor for cognitive impairments and dementia. This study examined the relationship between high-impact pain (pain that limits daily activities) and subjective cognitive decline (SCD) in 13,763 adults aged 50 and older from the Health and Retirement Study (2004–2020). High-impact pain was associated with a higher prevalence and incidence of SCD as compared to no pain and low-impact pain, adjusted for sociodemographic and clinical factors. Additionally, high-impact pain predicted an increased risk of objective cognitive impairment, particularly in individuals without the APOE4 allele. Our findings suggest that high-impact pain is a stronger predictor of future cognitive impairments than SCD alone in most of the population who do not carry the APOE4 allele. Interventions targeting high-impact pain, starting in middle age, may help mitigate the risk of cognitive decline and dementia. Future research is needed to understand potential mechanisms and develop effective cognitive aging strategies considering the impact of pain itself on cognition.

## Introduction

Chronic pain and cognitive decline are alarming health challenges for a rapidly growing aging population in the United States (U.S.).^1–4^ Chronic pain (i.e., pain that persists for ≥ 3 months) affects about a third of older adults, and up to half of those report high-impact pain, or pain that limits life and work activities.^5,6^ Similarly, 22–27% of older adults have mild cognitive impairment (MCI) and about 10% have dementia.^3,4^ Often, chronic pain and cognitive dysfunction overlap, and a growing body of evidence indicates that chronic pain increases the risk of Alzheimer’s disease and related dementias (ADRD).^7,8^ Indeed, whereas 21% of U.S. adults report chronic pain,^5^ its prevalence in people with ADRD approximates 50%.^9^ Individuals with chronic pain often display neurocognitive deficits in memory, attention, processing speed, and executive function.^7,10^ Moreover, our work and others have demonstrated that chronic pain is associated with neurobiological alterations, including accelerated brain aging, which in turn is associated with cognitive impairment and dementia.^11–15^ Nevertheless, there have been significant challenges to the study of pain’s impact on cognition, such as limitations in assessing the multidimensional pain experience using verbal reports in patients with dementia. As such, understanding the role of pain in the progression to ADRD before overt cognitive deficits occur may allow for targeted interventions to mitigate or prevent cognitive decline.

During the preclinical stage of AD, prior to the development of objective cognitive impairments (i.e., those measurable via cognitive testing), individuals may self-report losses in cognitive function, especially memory.^16^ This period in the cognitive trajectory is referred to as subjective cognitive decline (SCD) and is defined by: 1) self-reported decline in cognitive capacity that is unrelated to an acute event; 2) normal age-, sex-, and education-adjusted performance on standardized cognitive testing; 3) absence of MCI, prodromal AD or dementia; and 4) cannot be explained by a psychiatric or neurologic disease, medical disorder, medication, or substance use.^16^ Although not exclusive to AD, subjective cognitive decline (SCD) is a significant predictor of incident MCI and ADRD.^17^ Furthermore, brain- and AD-related biomarkers tend to be elevated in people with SCD and predict progression to AD.^18^ Consequently, there has been a growing interest in SCD as a target of early interventions to mitigate future cognitive impairments.^18^

Understanding the role of pain in the pre-clinical stage of ADRD may help develop interventions that mitigate the risk of cognitive decline in the high-risk group of older adults with chronic pain. Yet, current knowledge of pain and SCD is very limited. Recent studies have shown that chronic musculoskeletal pain is associated with a higher prevalence of SCD^19^ and persistent pain is associated with a higher risk of SCD development.^20^ However, these studies did not exclude objective cognitive impairments in their operationalization of SCD, as recommended.^16^ Furthermore, these studies did not evaluate pain-impact categories. Critically, high-impact chronic pain is not only associated with a greater pain burden but with accelerated aging processes as well. We and others have shown that high-impact pain is associated with several markers of accelerated aging, including telomere shortening,^21^ epigenetic aging,^22–24^ and brain aging,^25–27^ all of which correlate with cognitive function and decline. Although, to the best of our knowledge, no study to date has specifically evaluated high-impact pain in relation to cognition, pain interference (a measure of pain impact) has been associated with poorer cognitive performance and cognitive impairments.^28,29^ As such, individuals with high-impact pain may be at increased risk of developing SCD and progression toward objective cognitive impairments.

Therefore, we evaluated the relationship between high-impact pain and SCD, both cross-sectionally and longitudinally, and how these factors interact on the progression to objective cognitive impairments in a nationally representative sample of U.S. older adults. We hypothesized that individuals reporting high-impact pain would also report a higher prevalence of SCD with a higher risk of incident SCD, and that their co-occurrence would be associated with a higher risk of future development of objective cognitive impairments as compared to either high-impact pain or SCD alone.

## Methods

**Figure 1:**
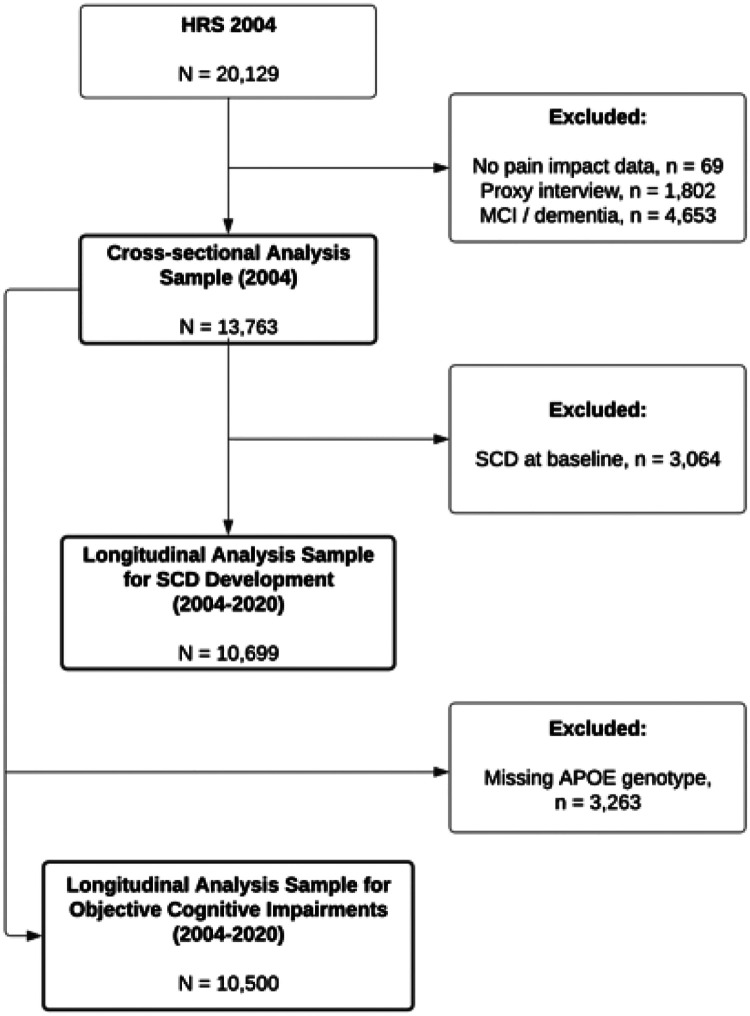
Participant Flowchart

This study utilized data from adults 50 years of age and older in the Health and Retirement Study (HRS) spanning the 2004–2020 waves. The HRS is a nationally representative biennial survey of older adults in the United States conducted by the University of Michigan. The 2004 wave was selected as the starting point for this analysis because a new cohort of participants, the Early Baby Boomers, was added to the HRS in that wave. Data were drawn from the RAND HRS Longitudinal File Version 2 and the 2004 HRS Core Interview.^30^ Fig. 1 shows the sample flowchart for the analytic sample. Out of 20,129 respondents in 2004, a total of 6,496 were excluded due to missing or invalid data on pain (n = 69), proxy interviews (n = 1,802), or objective cognitive impairments (n = 4,653). As such, the analytic sample resulted in 13,763 participants.

### Pain Impact

High-impact pain was operationalized via two questions fielded in nearly every HRS wave since its inception. Participants who reported often being troubled by pain and that pain makes it difficult to do their usual activities were categorized as having high-impact pain. Otherwise, those who reported pain without activity limitations were categorized as having low-impact pain. We have previously shown that this operationalization is consistent with greater pain severity plus self-reported and objective limitations in physical function.^22^

### Objective Cognitive Impairment

Cognitive status was assessed using the Langa-Weir Classifications and the researcher-contributed dataset by Langa et al.^31,32^ This dataset provides a comprehensive summary score for cognitive function based on measures from the core HRS interviews. The summary score is derived from a 27-point scale based on immediate and delayed 10-noun recall tests, serial sevens test, and counting backwards test. The Langa-Weir Classifications categorize cognitive status as normal, cognitive impairment without dementia (also known as mild cognitive impairment), and dementia. Objective cognitive impairment was defined as cognitive impairment with or without dementia.

### Subjective Cognitive Decline

Participants were asked to rate their memory compared to their prior interview (or two years ago) as better, about the same, or worse. Individuals classified as having a normal objective cognitive status as described above who also answered “worse” were classified as having SCD.

### Apolipoprotein E (APOE) genotype

The HRS has assessed APOE genotype from a large subset of participants who consented and completed salivary DNA collection between 2006 and 2012. We included genotype data for the rs7412 and rs429358 single nucleotide polymorphisms that define the ε4 allele. In total, the dataset includes 17,237 participants with directly genotyped APOE and 1,956 participants with imputed data. As recommended, we excluded imputed genotype data with a posterior probability less than 0.8 for either rs7412 or rs429358.^33^ Consistent with previous work^19^ and for simplicity in the models, we dichotomized our sample into individuals having no ε4 allele (i.e., ε3/ε3, ε3/ε2, ε2/ε2) and those carrying at least one ε4 allele (i.e., ε4/ε4, ε4/ε3, ε4/ε2).

#### Additional Explanatory Variables:

Chronic pain and cognitive decline share many risk factors, including biological, psychosocial, and lifestyle factors.^7^ We, therefore, include the following covariates in the analyses: age, sex, race (White/Caucasian, Black/African American, other), Hispanic ethnicity, education (less than high school, high school or GED, some college, bachelor’s degree, advanced degree), self-reported body mass index (kg/m^2^), alcohol consumption (never or rarely, moderate [1 drink/day for women, 1–2 drinks/day for men], excessive [≥ 2 drink/day for women, ≥ 3 drinks/day for men]), cigarette smoking (never, past smoker, current smoker), comorbidity index (based on the presence of eight diseases: high blood pressure, diabetes, cancer, lung disease, heart disease, stroke, psychiatric problems, and arthritis), and the 8-item Center for Epidemiologic Studies Depression Scale (CES-D-8).

##### Statistical Analysis:

All statistical analyses were conducted using SAS software (version 9.4). Descriptive statistics were performed, including ANOVA and Rao-Scott chi-square tests, and data are presented as raw counts with weighted percentages and weighted means with standard errors. For cross-sectional analyses, logistic regression models were employed to examine the association between pain impact and SCD. The PROC SURVEYLOGISTIC procedure was used to account for the complex survey design, including stratification, clustering, and sample weights. This procedure allows for the incorporation of survey design variables to produce unbiased parameter estimates and standard errors. Additionally, analyses were performed using Cox proportional hazards models to assess the relationship between pain impact and the time-to-event (in months) for SCD and objective cognitive impairments. The PROC SURVEYPHREG procedure was utilized to account for the survey design. We also used the cumulative incidence function (PROC LIFETEST) to visualize the time-to-event data across groups. For all models, we first used simple models with the primary independent and dependent variables, reported as odds ratios (OR) or hazard ratios (HR). Subsequently, we performed multivariable models adjusting for all covariates, reported as adjusted odds ratios (aOR) or adjusted hazard ratios (aHR). Missing values on race/ethnicity (n = 3), education (n = 3), self-reported BMI (n = 203), alcohol consumption (n = 25), smoking (n = 102), and CES-D-8 (n = 18) were considered missing at random and imputed via regression imputation. Since the APOE- 4-positive genotype does not reliably predict SCD development^34^ and data was missing on a large portion of the sample (n = 3, 263), it was not included in the analyses with SCD as the outcome. Nevertheless, we performed additional analyses including APOE-4 in the models, which can be found in the **Supplementary Materials.** Results were considered statistically significant at an alpha level of 0.05. To control for multiple comparisons, we applied the Benjamini-Hochberg method using a false discovery rate (FDR) set at 0.05 to maintain the statistical rigor while minimizing the risk of type I errors.

## Results

Participants were between 50 and 98 years old, with a mean age of 63.0 (SE = 0.2) years. In total, 68.3% reported no pain, 12.3% reported low-impact pain, and 18.9% reported high-impact pain. There were small but significant differences in characteristics between pain impact groups (Table 1). Compared to no pain or low-impact pain, individuals with high-impact pain were more likely to be female, have less than a high school education, have a higher BMI, currently smoke cigarettes, never or rarely consume alcohol, have a higher comorbidity index, and more depressive symptoms.

## High-Impact Pain is Associated with Higher Prevalence of Subjective Cognitive Decline

A total of 21.1% (n = 3,064) of participants reported SCD. The prevalence of SCD was significantly higher in high-impact pain (32.4%) compared to no pain (18.0%) or low-impact pain (21.3%); P < 0.0001, corrected P = 0.0001 (Table 1). As shown in Table 2, individuals with high-impact pain had significantly higher odds of SCD compared to those with no pain, with an OR of 2.17 (95% CI: 1.94, 2.44) in the univariate analysis and an aOR of 1.48 (95% CI: 1.30, 1.69) when controlling for demographics, education, BMI, alcohol consumption, smoking, comorbidity index, and depressive symptoms. Similarly, the odds were also higher in high-impact pain compared to low-impact pain, with an aOR of 1.32 (95% CI: 1.11, 1.57). Low-impact pain showed modestly higher odds of SCD versus no pain, but the association did not survive multivariable or multiple comparisons adjustment (P = 0.085). These associations remained unchanged when further adjusted for APOE4 status **(Supplementary Table 1),** which was not independently associated with SCD (P = 0.062; data not shown).

## High-Impact Pain is Associated with Higher Risk of Subjective Cognitive Decline

The median (interquartile range) time-to-event for SCD development were 197 (167, 205), 194 (147, 202), and 191 (123, 201) months for no pain, low-impact pain, and high-impact pain, respectively. In total, 87.0%, 83.3%, and 80.5% of events were censored for no pain, low-impact pain, and high-impact pain, respectively.

**Figure 2:**
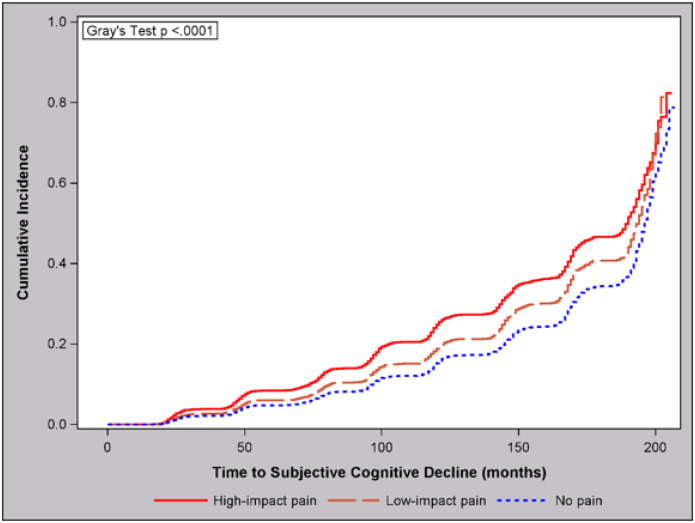
Cumulative Incidence of Subjective Cognitive Decline by Pain Impact Status (HRS 2004–2020, N = 10,699)

Following cross-sectional analysis, we evaluated the longitudinal association between high-impact pain and the development of SCD between 2004 and 2020 among those without SCD at baseline (Table 3, Fig. 2). Cox proportional hazards regression showed that individuals with high-impact pain had a significantly higher risk of SCD compared to those with no pain (HR = 1.62, 95% CI: 1.51, 1.73), and this relationship remained significant after controlling for additional explanatory variables (aHR = 1.31, 95% CI: 1.21, 1.42). Similarly, the risk of SCD was higher for high-impact pain as compared to low-impact pain (aHR = 1.18, 95% CI: 1.06, 1.32). The risk of SCD was also elevated for low-impact pain versus no pain, although again the association did not survive multivariable or multiple comparison adjustment (P = 0.10). As seen cross-sectionally, these relationships remained nearly unchanged after adjustment for APOE4 status **(Supplementary Table 2)**, which was not independently associated with SCD (P = 0.076; data not shown). A cumulative incidence plot can be seen in Fig. 2.

### High-Impact Pain is Associated with Higher Risk of Objective Cognitive Impairments in Persons who are APOE4 Negative

We then evaluated the longitudinal association between high-impact pain, SCD, and their interaction at baseline and the risk of developing objective cognitive impairments between 2004 and 2020. For this analysis, we restricted the analysis to participants with APOE genotype data (N = 10,500). In simple regressions, high-impact pain was associated with a similarly increased risk of objective cognitive impairments as compared to no pain (HR = 1.35, 95% CI: 1.23, 1.49) and low-impact pain (HR = 1.43, 95% CI: 1.24, 1.64), whereas no difference was observed between low-impact pain and no pain (P = 0.357).

**Figure 3:**
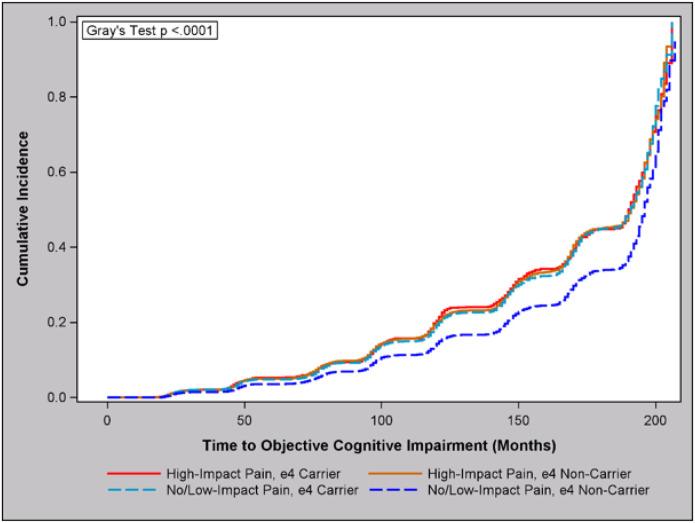
Cumulative Incidence of Objective Cognitive Impairment by Pain Impact Status, Stratified by APOE-4 Genotype (HRS 2004–2020, N = 10,699)

We found no significant interaction between pain impact and SCD (P = 0.46). On the other hand, we found a significant interaction effect between pain impact and APOE4 carrier status (P = 0.005). Given the results and the complexity of an interaction effect between two categorical factors, we collapsed the categories of low-impact pain and no pain into a single group to better illustrate the findings. The results, shown in Tables 4–5 and Fig. 3, show that high-impact pain was associated with a 52% (95% CI: 37–69%) increased risk of objective cognitive impairment as compared to having low-impact or no pain only in those who were negative for APOE4, and this relationship was maintained after adjustment for additional explanatory factors (aHR = 1.21, 95% CI: 1.07, 1.36). Inversely, APOE4 carrier status was associated with a 34% (95% CI: 24–45%) increased risk of objective cognitive impairment only in those with low-impact or no pain, and this relationship was maintained after multivariable and multiple comparison adjustment (aHR = 1.46, 95% CI: 1.36, 1.57).

## Discussion

We conducted the first examination of how high-impact pain (i.e., pain that limits daily activity) relates to subjective cognitive decline, which is a known symptomatic indicator of preclinical dementias, in a large nationally representative sample of older adults. Several important contributions emerged from this investigation. First, older individuals with high-impact pain had an increased prevalence and incidence of subjective cognitive decline. Second, high-impact pain was independently associated with an increased incidence of objective cognitive impairments in older adults, particularly those without the high-risk APOE4 polymorphism. Finally, the APOE4 genotype predicted cognitive impairment only in those without high-impact pain. Altogether, our results suggest that the effect of pain on cognitive decline in aging is largely driven by high-impact pain even after accounting for known risk factors. Given its high prevalence among older adults, high-impact pain appears to be a stronger predictor of future cognitive impairments than SCD in persons who do not carry the high-risk APOE4 allele, which is most of the population. These results suggest that interventions for high-impact chronic pain may help mitigate the risk of progressive cognitive decline and the development of future dementia.

An association between pain and cognitive impairments has long been observed and the relationship can be bidirectional.^7,8^ However, to our knowledge, this is the first study to report an association between high-impact pain and SCD. While other large-scale studies have reported associations between pain and SCD,^19,20^ we further excluded objectively measured cognitive impairments in the operationalization of SCD. Furthermore, this is the first study to specifically evaluate the risk of incident cognitive impairments in high-impact pain compared to low-impact pain. Prior epidemiological studies have shown that older adults with persistent pain demonstrate accelerated cognitive decline and are more likely to develop dementia.^20,35^ In other studies, cognitive decline has been dependent on greater pain intensity, number of pain sites, or pain interference.^36–40^ As a simple but multidimensional measure, high-impact pain is able to capture these individual pain domains at the population level. As such, our findings emphasize the importance of measuring high-impact chronic pain rather than unidimensional measures of pain duration, intensity, or interference alone. Indeed, high-impact chronic pain is considered a pain surveillance standard by the U.S. National Pain Strategy,^41^ but few large-scale surveys have implemented measures of the construct.^42^

The interaction between high-impact pain and APOE4 identified in this study has potential implications for the assessment of dementia risk and requires further investigation. To our knowledge, we are the first to report a moderation effect between APOE4 and pain impact on cognitive decline where high-impact pain predicts cognitive impairment only in persons who do not carry the APOE4 allele. Interestingly, Dhillon & Singh hypothesized that the APOE gene may indirectly influence pain modulation and pain outcomes, discussing its role in underlying processes, such as inflammation and neuronal signaling.42 Lending some validity to the hypothesis, Romano et al. observed lower sensitivity with similar pain intensity but greater unpleasantness to experimentally induced heat pain in cognitively unimpaired adults (median age 65 [46, 80] years) who were APOE4 positive (n = 12) compared to those who were APOE4 negative (n = 37).43 Given those findings, it is possible that lower pain sensitivity may account for a diminished role of high-impact pain on cognitive decline among APOE4-positive individuals. On the other hand, high-impact pain is not a reflection of sensitivity per se, but the overall burden of pain, including its intensity and daily interference with life.

Our finding that high-impact pain predicted cognitive impairment only in those without the APOE4 genotype further confirms previous work on the severe impact of APOE4 positivity in the higher risk of developing future cognitive decline.^43^ However, another possibility exists. It is known that in APOE4 negative individuals, vascular factors may have a more important role. It is plausible that high-impact pain reduces activity levels, as seen with increased BMI and worse metabolic factors. Thus, the interaction seen here may reflect how high-impact pain reduces health behaviors which mitigate cognitive impairment risk in people otherwise at reduced risk for cognitive impairment. Altogether, our results suggest that the effect of pain on cognitive decline in aging is largely driven by high-impact pain even after accounting for known risk factors. Given its high prevalence among older adults, high-impact pain appears to be a stronger predictor of future cognitive impairments than SCD in persons that do not carry the high-risk APOE4 allele, which is the majority of the population. Future studies with more sensitive multimodal assessments of pain may be able to more comprehensively elucidate the relationship between APOE4 positivity and high-impact pain.

Our findings are potentially impactful for clinical practice given the limited effective treatments for cognitive impairments once they have developed. This has contributed to a growing interest in SCD as an early target for interventions to mitigate the risk of ADRD. Similarly, there are limited effective treatments for debilitating chronic pain, and the use of opioids increases the risks of polypharmacy among older adults. Additionally, assessing pain in people with cognitive impairments is challenging due to the impact on their self-reporting, adding difficulty to pain management therapies. Furthermore, individuals with comorbid chronic pain and cognitive impairment, particularly those with high-impact pain, are more likely to report poorer health, more significant social and functional limitations, increased disability, and higher healthcare usage compared to those with only chronic pain or cognitive impairment.^44^ As such, the co-existence of chronic pain and cognitive impairments seems to indicate a poor prognosis.

Several strengths and limitations of this study should be acknowledged. The study utilized a large nationally representative sample of older adults in the U.S. The presence of cognitive impairments was determined with objective neuropsychological assessments, strengthening the operationalization of SCD and cognitive decline. Nevertheless, the large HRS sample necessitated the use of quick and easy-to-administer cognitive assessments, and future research with more comprehensive cognitive batteries may be able to better elucidate the relationships observed in this study. Furthermore, we used an operationalization of high-impact pain that has shown concurrent validity with subjective measures of pain intensity and disability, as well as objective measures of physical performance.^22^ That said, future studies using multimodal assessments of pain are needed. To be noted, the HRS questions used to operationalize pain-impact do not contain a duration component. As such, we do not refer to high-impact chronic pain in the interest of caution. Nevertheless, nearly three-quarters of individuals with high-impact pain in the HRS report pain durations lasting 2 to 3 months or longer (72.3%) and continue to report high-impact pain after 2 years (69.4%) (Tamargo, et al; Unpublished).

Despite extensive research efforts dedicated to cognitive decline in aging, the impact of pain on the brain and cognition has been understudied. The observed associations are potentially partly due to neuroplastic adaptations that are particularly pronounced in high-impact chronic pain and can alter cognitive function and brain health. Prospective studies are needed that examine how high-impact chronic pain may lead to brain changes and their association with subjective and objective cognitive decline. Fortunately, both high-impact chronic pain^5^ and SCD are simple, inexpensive measures that can be easily implemented in clinical settings and large-scale surveys. Furthermore, pain management guidelines have largely focused on the impact of opioids on cognition, but future practice should consider the impact of pain itself.

## Conclusion

This study highlights a significant association between high-impact pain and increased risk of subjective cognitive decline and objective cognitive impairments in older adults. High-impact pain may be a stronger predictor of future cognitive impairments than subjective cognitive decline, currently considered an important pre-clinical risk factor for dementia. Interventions targeting high-impact chronic pain may help mitigate the risk of progressive cognitive decline and the development of Alzheimer’s disease and related dementias. Future research is needed to understand potential mechanisms, such as neuroplastic adaptations due to high-impact pain, and to develop effective cognitive aging strategies that consider the impact of pain itself on cognition.

## Figures and Tables

**Table 1:** Sample Characteristics HRS 2004 (N = 13,763)

		No Pain	Low-Impact Pain	High-Impact Pain	P-value	Corrected P-value^a^
		% (SE)	% (SE)	% (SE)		
		Mean (SE)	Mean (SE)	Mean (SE)		
N (weighted %)		9,454 (68.3)	1,721 (12.3)	2,588 (18.9)		
Age	50+	63.1 (0.2)	62.7 (0.3)	62.8 (0.3)	0.268^b^	0.292
Sex	Female	53.6 (0.6)	55.3 (1.5)	64.6 (1.0)	< 0.0001^c^	0.0001
Race	White/Caucasian	86.5 (0.9)	90.5 (5.6)	88.7 (0.5)	0.012^c^	0.016
	Black/African American	8.8 (0.7)	5.6 (0.7)	7.4 (0.4)		
	Other	4.7 (0.6)	3.9 (0.7)	3.9 (0.3)		
Hispanic		5.4 (0.6)	6.5 (1.1)	5.6 (0.9)	0.297^c^	0.297
Education	Less than High-School	10.2 (0.5)	11.5 (1.1)	16.5 (1.0)	< 0.0001^c^	0.0001
	GED	3.3 (0.2)	5.0 (0.8)	7.0 (0.6)		
	High-School	30.2 (0.6)	31.7 (1.3)	31.8 (1.1)		
	Some college	25.1 (0.6)	28.1 (1.5)	27.0 (1.0)		
	College and above	31.2 (0.9)	23.8 (1.7)	17.7 (1.03)		
Body mass index	kg/m^2^	27.1 (0.1)	28.1 (0.2)	29.7 (0.2)	< 0.0001^b^	0.0001
Alcohol	Never or rarely	56.7 (0.9)	61.3 (1.6)	71.8 (1.3)	< 0.0001^c^	0.0001
	Moderate	27.0 (0.7)	23.3 (1.3)	15.9 (1.0)		
	Excessive	16.3 (0.6)	15.4 (1.0)	12.3 (0.9)		
Cigarette smoking	Never	44.3 (0.7)	40.0 (1.5)	37.9 (0.9)	< 0.0001^c^	0.0001
	Past	40.4 (0.6)	45.0 (1.4)	41.8 (1.4)		
	Current	15.3 (0.6)	15.1 (0.9)	20.3 (1.2)		
Comorbidity Index	0–11	1.3 (0.02)	1.7 (0.03)	2.4 (0.03)	< 0.0001^b^	0.0001
CES-D	0–8	0.9 (0.02)	1.4 (0.05)	2.7 (0.07)	< 0.0001^b^	0.0001
APOE	4 positive	26.3 (0.7)	23.4 (1.4)	27.5 (0.9)	0.074^c^	0.089
SCD		18.0 (0.5)	21.3 (1.0)	32.4 (1.1)	< 0.0001^c^	0.0001

a False discovery rate-adjusted p-value

b ANOVA

c Rao-Scott Chi-square

**Table 2:** Logistic Regressions for Subjective Cognitive Decline (N = 13,763)

	Univariate^a^ OR (95% CI)	P-value	Multivariable^b^ aOR (95% CI)	P-value	Corrected P-value^c^
High-impact pain vs no pain	2.17 (1.94, 2.44)	< 0.0001	1.47 (1.30, 1.67)	< 0.0001	0.0003
High-impact pain vs low-impact pain	1.77 (1.50, 2.08)	< 0.0001	1.32 (1.11, 1.57)	0.003	0.0045
Low-impact pain vs no pain	1.23 (1.09, 1.39)	0.001	1.11 (0.99, 1.26)	0.085	0.085

a Adjusted for sample weights and stratified cluster design

b Additionally adjusted for demographics (age, race, Hispanic ethnicity, sex), education (years), BMI, alcohol consumption, cigarette smoking, comorbidity index, and depressive symptoms; (N = 13,418)

c False discovery rate-adjusted p-value

**Table 3:** Cox Proportional Hazards Regressions for Subjective Cognitive Decline (N = 10,699)

	Univariate^a^ HR (95% CI)	P-value	Multivariable^b^ aHR (95% CI)	P-value	Corrected P-value^c^
High-impact pain vs no pain	1.62 (1.51, 1.73)	< 0.0001	1.31 (1.21, 1.42)	< .0001	0.0003
High-impact pain vs low-impact pain	1.28 (1.13, 1.45)	0.0002	1.18 (1.06, 1.32)	0.004	0.006
Low-impact pain vs no pain	1.27 (1.13, 1.42)	0.0002	1.11 (0.98, 1.25)	0.101	0.101

a Adjusted for sample weights and stratified cluster design

b Additionally adjusted for demographics (age, race, Hispanic ethnicity, sex), education, comorbidity index, and depressive symptoms; (N = 10,421)

c False discovery rate-adjusted p-value

**Table 4:** Cox Proportional Hazards Regressions for Objective Cognitive Impairment (N = 10,500)

	Univariate^a^ HR (95% CI)	P-value	Multivariable^b^ aHR (95% CI)	P-value	Corrected P-value^c^
High-impact pain vs low-impact/no pain	1.39 (1.28, 1.51)	< 0.0001	1.13 (1.01, 1.26)	0.034	0.068
SCD vs no SCD	1.23 (1.13, 1.33)	< 0.0001	1.02 (0.94, 1.10)	0.629	0.629
APOE4 carrier	1.24 (1.15, 1.35)	< 0.0001	1.40 (1.31, 1.50)	< 0.0001	0.0003

a Adjusted for sample weights and stratified cluster design

b Additionally adjusted for demographics (age, race, Hispanic ethnicity, sex), education, comorbidity index, depressive symptoms, and APOE4 status

c False discovery rate-adjusted p-value

**Table 5:** Cox Proportional Hazards Regressions for Objective Cognitive Impairment (N = 13,763)

Predictor	Strata	Univariate^a^ HR (95% CI)	P-value	Multivariable^b^ aHR (95% CI)	P-value	Corrected P-value^c^
High-impact vs low-impact/no pain	APOE4 negative	1.52 (1.37, 1.69)	< 0.0001	1.21 (1.07, 1.36)	0.003	0.006
High-impact vs low-impact/no pain	APOE-4 positive	1.03 (0.84, 1.26)	0.803	0.95 (0.78, 1.15)	0.558	0.558
APOE4 positive vs negative	Low-impact/no pain	1.34 (1.24, 1.45)	< 0.0001	1.46 (1.36, 1.57)	< 0.0001	0.0003
APOE4 positive vs negative	High-impact pain	0.90 (0.72, 1.13)	0.373	1.15 (0.96, 1.37)	0.122	0.163

a Adjusted for sample weights and stratified cluster design

b Additionally adjusted for demographics (age, race, Hispanic ethnicity, sex), education, comorbidity index, depressive symptoms, and APOE4 status

c False discovery rate-adjusted p-value
